# An Autistic Endophenotype and Testosterone Are Involved in an Atypical Decline in Selective Attention and Visuospatial Processing in Middle-Aged Women

**DOI:** 10.3390/ijerph121215033

**Published:** 2015-12-15

**Authors:** Romero-Martínez Ángel, Moya-Albiol Luis

**Affiliations:** Department of Psychobiology, University of Valencia, Avenida Blasco Ibañez, 21, 46010 Valencia, Spain; luis.moya@uv.es

**Keywords:** Autism spectrum disorders, caregiver, selective attention, testosterone, women

## Abstract

Mothers of offspring with autism spectrum disorders (ASD) could present mild forms of their children’s cognitive characteristics, resulting from prenatal brain exposure and sensitivity to testosterone (T). Indeed, their cognition is frequently characterized by hyper-systemizing, outperforming in tests that assess cognitive domains such as selective attention, and fine motor and visuospatial skills. In the general population, all these start to decline around the mid-forties. This study aimed to characterize whether middle-aged women who are biological mothers of individuals with ASD had better performance in the aforementioned cognitive skills than mothers of normative children (in both groups *n* = 22; mean age = 45), using the standardized Stroop and mirror-drawing tests. We also examined the role of T in their performance in the aforementioned tests. ASD mothers outperformed controls in both tests, giving more correct answers and making fewer mistakes. In addition, they presented higher T levels, which have been associated with better cognitive performance. Cognitive decline in specific skills with aging could be delayed in these middle-aged women, corresponding to a cognitive endophenotype, T playing an important role in this process.

## 1. Introduction

The existence of an autism spectrum disorder (ASD) endophenotype and the high heritability of this disorder could explain why ASD parents present mild forms of cognitive characteristics that are fully developed in their offspring. Like their offspring, ASD parents tend to present communication and cognitive flexibility deficits. Nevertheless, due to their highly systemizing minds they outperform or at least have similar scores to parents of non-affected children in certain cognitive abilities, such as selective attention and visuospatial processing [1,2,3,4]. These abilities and also psychomotor skills become impaired with aging [5,6,7], the decline starting around the mid-forties [8]. Nevertheless, the effect of aging on these cognitive skills has not been analyzed in first-degree relatives of people with ASD such as their biological mothers.

In parallel with cognitive decline, testosterone (T) levels drop with aging [9]. Moreover, the administration of exogenous T and consequent increase in T levels in young and elderly women improves processing speed, selective attention, and fine motor and visuospatial skills [10,11,12]. Several authors have indicated that chronically stressed middle-aged women have higher T levels, including mothers of people with ASD compared to non-stressed women [13,14]. There is, however, a gap in the literature concerning the role of T in cognition in ASD mothers.

Given the complexity of this issue, as a first approach, we decided to investigate the performance of mothers of offspring with ASD on selective attention and fine motor and visuospatial processing speed tests. We aimed to investigate the existence of the ASD endophenotype and the effects of T levels on the performance of middle-aged ASD mothers in these cognitive domains. For this purpose, we tested ASD mothers and matched control women using two tests, the Stroop test and a mirror-drawing test. Based on the fact that ASD mothers present highly systemizing minds, we hypothesized that they would not present the decline in cognitive function in these domains that typically occurs with aging, showing better performance than controls [1,2,3,4,15]. We also examined the effects of T on these cognitive tasks. We expected to find that a higher baseline T and stronger T response to the tasks would be associated with better performance in these tasks, due to the positive association between T and these abilities in women [10,11,12].

## 2. Method

### 2.1. Participants

The final sample was composed of 46 participants, including 22 mothers of individuals with ASD and 22 mothers of individuals with typical developing (with no neurodevelopmental or psychiatric conditions).

ASD mothers were recruited from an association of parents of people with autism located in Valencia, Spain (*Asociación Valenciana de Padres de Autistas*). They participated voluntarily in the study and gave written informed consent in accordance with ethical guidelines for human research (Declaration of Helsinki). The ages of the offspring with ASD ranged from five to 30 years (14.62 ± 6.90), with a sex ratio of 20 males to two females, which is attributable to the high prevalence of this disorder in males. Offspring were clinically diagnosed with ASD by clinical staff following the DSM-5 criteria. Moreover, the majorities of the ASD offspring are highly dependent on their parents and live at home with their parents. Furthermore, those individuals with ASD never work and they also require lifelong care. The inclusion criterion for participating in the study was being a biological mother of an individual with a clinical diagnosis of an ASD. An adequate control group was selected: this was composed of mothers in the same age-range who had offspring without autistic traits and no chronic illness. Furthermore, in the control group, the number of offspring per mother and the mean age of the children were similar to that in the other group.

### 2.2. Procedure

Participants were instructed to abstain from eating, drinking stimulants (such as tea, coffee, or alcohol), brushing their teeth, and smoking during the 2-h period before arriving at the laboratory. The experimental procedure was performed between 4:00 p.m. and 7:00 p.m. to minimize hormonal variations attributable to the circadian rhythm, and each session lasted approximately 2.5 h. On arrival, participants’ anthropometric data (age, weight, height) were collected, compliance with the instructions was checked, and an initial saliva sample was collected to measure baseline testosterone levels. Participants were then taken to a different room that was soundproofed and kept at a constant temperature (22 ± 1 °C) for the neuropsychological tests. After a 5-min habituation period and a brief introduction to the tests, the participants remained silent for 3 min and then the next saliva sample was collected (to assess preparatory period T levels). All participants spent around the same period of time completing the Stroop and mirror-drawing tests (25–30 min). Immediately after completing these tests, a further saliva sample was collected (for post-test T levels). The participants then returned to the first room where additional saliva samples were collected at 20 and 30 min (for assessing T during recovery).

### 2.3. Neuropsychological Tests

#### 2.3.1. The Stroop Test

The Spanish version of the Stroop Color-Word Test (Stroop test) [16] was used to assess the participants’ ability to “resist interference”. They were instructed to name the ink color of the printed words while ignoring distracting stimuli (color name). The number of correct responses given in 45 s in the color-word condition was recorded as the dependent variable, as recommended in recent research [17,18]. Errors were indicated by the examiner, and participants were asked to correct them before continuing as quickly and as accurately as possible.

#### 2.3.2. The Mirror-Drawing Test

Mirror-drawing tests consist of tracing a shape that can only be seen inverted in a mirror. Specifically, in the test used, a mirror is mounted on a vertically orientated metallic support, while another support prevents participants from having a direct view of the model, the outline of a star. Every time there is a divergence from the line of the star, a short beep sounds indicating that the participant has made a mistake. Participants were allowed one practice circuit and instructed to prioritize accuracy over speed. The test needs multi-process agreement because the reversed visual feedback occurs concurrently with the process of visual-proprioceptive recalibration [19], and, hence, high levels of attention are necessary [20]. We used the number of traces (to the nearest ¼ of a circuit) completed in 120 s and errors as dependent variables.

### 2.4. Hormone Measurements

Saliva was directly collected from the mouth to a glass tube for T measurements. Participants were informed about the need to closely follow the instructions for saliva sampling in order to obtain valuable data. The samples were frozen at −20 °C until analysis by enzyme immunoassay using a T saliva Elisa kit (Diagnostics Biochem Canada Inc.: Dorchester, ON, Canada). The assay sensitivity was 1 pg/mL and results were expressed in pmol/L. Good precision was obtained, with intra- and inter-assay variation coefficients of 3.98% and 7.98%, respectively.

### 2.5. Data Analysis

After confirming the normality of the data using the Kolmogorov-Smirnov test, *t*-tests were performed with “group” as the between-subject factor for anthropometric data (age and body mass index) and cognitive test scores. Chi-square statistics were calculated for analyzing the frequencies of the demographic variables.

Magnitude of T response was estimated by the area under the curve with respect to the increase (AUCi), using the trapezoidal rule [21]. AUCi is calculated with reference to the baseline measurement, ignoring the distance from zero for all measurements and emphasizing the changes over time.

Spearman’s or Pearson’s correlations were calculated to assess relationships between variables as appropriate for each group (ASD mothers and controls).

Data analyses were carried out using IBM SPSS (Version 22.0, IBM: New York, NY, USA). Statistical significance was defined as *p* values ≤ 0.05; while there was considered to be a tendency to significance with *p* values from > 0.05 to *p* ≤ 0.07. Average values are reported in the tables as mean ± SD.

## 3. Results

Descriptive characteristics and cognitive performance on the neuropsychological tests of both ASD mothers and controls are summarized in Table 1. The groups did not differ in age, body mass index, menstrual cycle phase, marital status, educational level, number of children, smoking status or drug use. Moreover, a significant “group” effect was found in the AUCi for T (F_1,44_ = 4.20, *p* = 0.04, gp 2 = 0.10). In fact, though baseline T levels were not significantly different, T levels increased in ASD mothers but fell in controls.

A significant “group” effect was found in the “Stroop test correct responses”, “mirror-drawing test number of traces” and “mirror-drawing test number of mistakes” *t*_44_ = 2.39, *p* = 0.021, *d* = 0.72; *t*_44_ = 2.16, *p* = 0.036, *d* = 0.65, and *t*_44_ = −2.50, *p* = 0.016, *d* = 0.75, respectively. ASD mothers obtained more correct responses and traces in the Stroop test and mirror-drawing test and fewer errors in the mirror-drawing test than controls.

Relationships of T with Stroop test and mirror-drawing test performance for ASD mothers and controls are summarized in Table 2.

**Table 1:** Mean ± SD of age and body mass index (BMI) and demographic variables for mothers with autism spectrum disorders (ASD) offspring (ASD mothers) and controls.

Demographic Variables	ASD Mothers	Controls
**Age**	45.27 ± 1.71	45.00 ± 0.90
**BMI**	26.69 ± 0.99	25.37 ± 1.13
**Number of children**	1.92 ± 0.82	2.18 ± 0.90
**Number of children at home**	1.71 ± 0.75	2.14 ± 0.88
**Phases of menstrual cycle**		
Luteal (Day 1–14)	45.4%	33.4%
Follicular (Day 15-menstrual period)	36.4%	45.8%
Amenorrhea (>6 months)	18.2%	20.8%
**Educational level**		
≤12 years	32%	23%
University degree(s)	68%	77%
**Marital status**		
Married/Cohabiting	82%	86%
Divorced/Widowed/Single	18%	14%
**Employment status**		
Employed	95%	95%
Unemployed	0%	0%
Retired/Other	5%	5%
**Smoker**		
Yes	21%	40%
No	79%	60%
**Drug use (medical)**		
No	54%	36%
Yes	46%	64%
**Care recipient characteristics and caregiving situation**		
Autism-spectrum quotient (AQ adolescent)—degree of autism	31.75 ± 4.73	
Time caregiving since definitive Diagnosis (years)	9.73 ± 1.03	
Global activity	49.23 ± 5.37	
Independence (Barthel Index)	84.17 ± 3.61	
Disability percentage	38.85 ± 8.27	
Time caring per week (h/week)	62.13 ± 45.34	

**Table 2:** Pearson correlations between testosterone (T) and cognitive performance for mothers of ASD offspring and controls. * *p* < 0.05, ** *p* < 0.01.

	Stroop Test		Mirror Drawing Test Number of Traces		Mirror Drawing Test Errors	
	ASD	Controls	ASD	Controls	ASD	Controls
Baseline	0.036	0.019	0.288	0.196	0.056	0.022
AUC_i total_	0.088	0.021	0.031	0.098	0.033	0.131
AUC_i preparatory_	0.558 **	0.044	0.469 *	0.058	0.239	0.055

## 4. Discussion

ASD mothers gave more correct responses in the Stroop test and completed more traces in the mirror-drawing test, and further, they committed fewer mistakes in the mirror-drawing test than controls. In addition, an analysis of the relationship between T and cognitive performance revealed that a large increase in T levels in the preparatory period was associated with better performance in both tests.

Studies with ASD mothers have suggested that they present highly systemizing minds, outperforming their peers in certain tasks, particularly those which need good fine motor and visuospatial skills [14,22,23,24]. Our results support this hypothesis, in that, ASD women outperformed controls in the Stroop and mirror-drawing tests, demonstrating higher selective attention, better fine motor and visuospatial skills and processing speed than controls.

On the other hand, we should underline that ASD mothers of this study are caregivers of their offspring for a long period of time. That is, they are under chronic stress, and this has been associated with an acceleration of cognitive decline in processing speed and attention [25,26,27]. However, the healthy caregiver hypothesis suggests that caregivers may maintain their cognitive abilities, because caregiving requires engagement in tasks that are cognitively complex such as managing medications, arranging appointments and transportation, and juggling conflicting demands and this is consistent with our results; that is, exercising and challenging cognitive skills seem to slow the rate of cognitive decline [28]. Further research is needed, however, to clarify the specific components of cognition that are impaired or preserved.

It has also been suggested that high T levels may help maintain cognitive skills in both young and elderly women [10,11,12]. Our data is congruent with this hypothesis, in that, large increases in T before the test were associated with better performance in ASD mothers. Moreover, ASD mothers had higher overall T levels than controls and this could explain why they outperformed controls in these specific skills. These findings imply that the caregiving role may reduce the cognitive decline with aging in this population by triggering higher T levels, and this could be understood as a mechanism to safeguard the wellbeing of the care recipient. As chronic stress tends to be associated with high cortisol (C) levels, which tends to show deleterious effects on cognition, it would be also interesting to analyze the role of C in this kind of population. Moreover, the T maintains an inverse relationship with the C. Hence, future studies should consider the relationship between those hormones and their role on human cognition.

The main limitation of this study is that it is cross-sectional, and individual differences may mask other effects assessed at a single point in life. Moreover, we should recognize that it is exploratory in nature with the small sample size. It is, however, extremely difficult to conduct longitudinal studies of caregivers, given their caregiving commitments. Nonetheless, our data are relevant given the growing need for caregiving, and they are novel, as no laboratory studies have analyzed the T response to selective attention and visuospatial skill tests in middle-aged female caregivers of individuals with ASD.

## 5. Conclusions

In conclusion, this study demonstrates that cognitive decline with aging in middle-aged women who have children with ASD could be delayed or reduced, specifically in processing speed, selective attention, and fine motor and visuospatial skills by the ASD cognitive endophenotype and/or their systemizing minds. Moreover, it seems that T may play an important role in this process. Hence, it is essential to highlight the existence of this cognitive endophenotype characterized by high cognitive sensitivity to T effects and to develop a neuropsychological general assessment to check which domains should be targeted by interventions in this population.
